# Impact of Community-Based HIV/AIDS Treatment on Household Incomes in Uganda

**DOI:** 10.1371/journal.pone.0065625

**Published:** 2013-06-20

**Authors:** Joseph F. Feulefack, Martin K. Luckert, Sandeep Mohapatra, Sean B. Cash, Arif Alibhai, Walter Kipp

**Affiliations:** 1 Department or Resource Economics and Environmental Sociology, University of Alberta, Edmonton, Alberta, Canada; 2 Department of Food and Nutrition Policy, Friedman School of Nutrition Science and Policy, Tufts University, Boston, Massachusetts, United States of America; 3 Department of Public Health Sciences, School of Public Health, University of Alberta, Edmonton, Alberta, Canada; UNAIDS, Switzerland

## Abstract

Though health benefits to households in developing countries from antiretroviral treatment (ART) programs are widely reported in the literature, specific estimates regarding impacts of treatments on household incomes are rare. This type of information is important to governments and donors, as it is an indication of returns to their ART investments, and to better understand the role of HIV/AIDS in development. The objective of this study is to estimate the impact of a community-based ART program on household incomes in a previously underserved rural region of Uganda. A community-based ART program, based largely on labor contributions from community volunteers, was implemented and evaluated. All households with HIV/AIDS patients enrolled in the treatment programme (n = 134 households) were surveyed five times; once at the beginning of the treatment and every three months thereafter for a period of one year. Data were collected on household income from cash earnings and value of own production. The analysis, using ordinary least squares and quantile regressions, identifies the impact of the ART program on household incomes over the first year of the treatment, while controlling for heterogeneity in household characteristics and temporal changes. As a result of the treatment, health conditions of virtually all patients improved, and household incomes increased by approximately 30% to 40%, regardless of household income quantile. These increases in income, however, varied significantly depending on socio-demographic and socio-economic control variables. Overall, results show large and significant impacts of the ART program on household incomes, suggesting large returns to public investments in ART, and that treating HIV/AIDS is an important precondition for development. Moreover, development programs that invest in human capital and build wealth are important complements that can increase the returns to ART programs.

## Introduction

In sub-Saharan Africa, HIV/AIDS continues to be a major health threat, pushing communities, households and individuals over the brink of poverty and perpetuating poverty traps in unprecedented ways [1], [2], [3], [4]. The impact of the pandemic is particularly pronounced in rural communities, far from existing medical resources and support, and for those who are more vulnerable to a downward spiral of poverty [5].

Highly active antiretroviral therapy was first introduced in 1996, but by 2004, only 7 percent of those who needed treatment in developing countries were actually receiving treatment [6]. Between 2003 and 2008, access to ART programs increased by nearly thirty times to reach 2.9 million patients in sub-Saharan Africa [7]. Despite this progress, by 2008, only 44% of the 6.7 million HIV/AIDS patients in Africa had access to treatment [7], partially because of costs of treatment, difficulties of administering treatments in rural areas of countries with inadequate medical facilities and infrastructure challenges that make it difficult for patients to reach those resources that are available. To fill the gaps in the availability of broad based treatment, several initiatives led by governments, NGOs and aid organizations have been launched [8], [9]. But more recently, international funding has begun to decline, leaving developing countries to carry more of the financial burden to support ART programs [10].

The case for investing in ART programs seems intuitively clear, as a number of studies have investigated beneficial effects of treatments. Some studies have focussed on reductions of mortality in response to ART. For example, in Uganda, mortality decreased by 92% through the provision of ART drugs to HIV infected individuals [11]. Other studies have shown how labour productivity increases with ART in terms of reduced absenteeism, increased output, and participation in the workforce (e.g. [12], [13], [14]).

Despite these clearly beneficial effects, there is surprisingly little research that has investigated the economic impact of HIV/AIDS, and subsequent treatments. Of these few studies, most focus on the impacts of HIV/AIDS on infected households compared to non-infected households. Evidence in Kenya [15] and Zimbabwe [16] suggest that HIV/AIDS reduces household production by approximately one- to two-thirds, depending on the region, mix of crops, and socio-economic features of the household. Income of the poorest is often the worst affected by the adverse effects of HIV/AIDS [17], [18]. However, not all studies find a significant reduction in production due to HIV/AIDS. In a cross-country study in Africa [19], the impacts of HIV/AIDS on economic growth are not clear, especially considering areas where the level of HIV/AIDS prevalence is relatively high. Likewise, a study in Zambia finds no significant impact on per capita income associated with the death of a prime age adult from HIV/AIDS [20].

There are even fewer studies that have estimated the economic impact of ART programs on household livelihoods. To our knowledge, there is only one study that has quantified the effects of ART on household income in Africa. In South Africa, there was an 18-month evaluation of the impact of ART on 249 patients and their households living in the urbanized area of Soweto [21]. Cash income of ART patients’ households increased by 10 percent over a period of approximately 15 months, largely due to changes in employment opportunities that increased as the health of the patients improved. But such increases are likely to vary substantially across countries and between households. For example, in South Africa, access to social grants can potentially impact the amount of labour that households choose to supply [22]. Moreover, studies in Kenya have shown that households are likely to use resources freed by ART in different ways. Though some labour may be reallocated to increasing income, other resources may be reallocated to non-remunerating activities such as leisure [12], [23].

The absence of empirical evidence to support the benefits of ART programs in terms of household economic measures leaves governments with little information regarding how their health care investments influence the economic development of their countries. In particular, for those remote parts of Africa where ART coverage is least developed, there are no studies, to our knowledge, that have estimated the benefits of ART regarding their influences on household income. The objective of this paper is to contribute to the literature on benefits of ART programs by estimating impacts of treatments on household incomes in a rural area of sub-Saharan Africa. More specifically, we examine the effects of participation in a community-based HIV/AIDS treatment program on household income using a set of panel data from rural Uganda, while controlling for other factors (e.g. socio-demographic and geographic characteristics). Our analysis is based on a unique data set from rural Uganda that contains information on cash and subsistence income derived by rural households over one year of an ART program.

In the next section we discuss a number of aspects regarding methods. First we describe the study site, the treatment programme and data collection. We then explain our empirical approach by specifying an econometric model, describing how variables are constructed, and presenting our estimation approach. Results are presented in the next section, followed by our conclusions.

### Methods

Our empirical approach focuses on estimating the average effect of treatment on household incomes while paying special attention to heterogeneity in the estimated impact. To this end we first specify a regression model that yields estimates of the mean impact of treatment on incomes. These estimates can be regarded as the impact of treatment for the average individual. Second, we use quantile regressions to identify how the effect of ARV treatments can change across the income distribution. The latter set of estimates will be especially useful from a policy perspective since they will shed light on the differential effects of ARV treatments on individuals who are receiving treatment, but are living in households with different income levels.

### The Study Site, the Treatment Program and Data Collection

The study site is a subcounty located in the Kabarole district of western Uganda. Household members in Kabarole district earn their livelihoods mainly from agriculture through involvement in cropping, animal rearing and/or plantation industry jobs. HIV/AIDS prevalence in the area is estimated to be 11%, one of the highest in the country [24], [25].

The data for this study were generated as part of a community-based ART program for HIV/AIDS patients, undertaken by the University of Alberta in conjunction with the Kabarole District Health Department, Uganda. Ethics approval was provided in Canada by the Health Research Ethics Board, University of Alberta, Edmonton, and in Uganda by the Uganda National Council of Science and Technology, Kampala, the School of Public Health, Makerere University, Kampala and the District Health Officer for Kabarole District. Each participant was informed about the study and provided written consent through a signature or a thumbprint.

Patients were enrolled in the treatment programme if they matched five eligibility criteria: (1) residency in the sub-county, (2) age of eighteen years or more at the initiation of treatment, (3) treatment naive, (4) qualification for ART following Uganda ART therapy guidelines (i.e. CD4 cell count <200 cells/mm3 or World Health Organization clinical stage 3 or 4), and (5) willingness of the patient to accept daily treatment support by family/friends and to receive weekly visits from a community volunteer.

Information about the treatment programme was dispersed through three primary means: 1) health workers at the health centre that were seeing patients that displayed AIDS symptoms told the patients about the program; 2) posters were used at and around the clinic, and 3) word of mouth. A total of 185 patients were started on treatment between March, 2006 and June, 2007. After 2 years on treatment, clinical outcomes of the community-based patients were comparable to those of a best-practice urban hospital in the same district [26]. The health related quality of life of the community-based patients also improved significantly [27].

This study uses panel data collected with an in-depth household livelihood survey conducted among patients registered in the ART programme. The surveys were based in part on instruments developed by the Poverty and Environment Network (PEN; http://www.cifor.org/pen/research-tools/the-pen-prototype-questionnaire.html) of the Centre for International Forestry Research (CIFOR). The survey is comprised of two main parts: (1) the baseline household survey questionnaire and (2) the quarterly survey. The baseline survey was designed to capture some basic characteristics of the household, whereas the quarterly survey was designed to track seasonally other variables of interest, such as income, throughout a year-long period. Each survey round included extensive questions on household activities, possessions, and household member time use. Quarterly surveys were administered 5 times in order to capture 1 quarter before treatment and 4 quarters after treatment began. Enumerators were all fluent in English and in Rutooro, the local language, and underwent a full week of training prior to conducting surveys. They administered the baseline household survey shortly after patients were enrolled in the program. A total of 185 patients were initially enrolled in the treatment program. Of these patients, 163 patient households took part in the first interview, because some patients died prior to the start of the household survey and some patients could not be located based on the information that they provided. The number of households dropped further to 134 by the fifth visit. For our analysis, we use data from the 134 households over 5 visits, providing a total of 670 observations.

### The Empirical Approach

#### Model specification

Our objective in this section is to estimate the impact of ART programs on households’ income using a multivariate regression model. Regression analysis focuses on the effect of explanatory variables on the mean of the conditional distribution of the dependent variable. However, if ART affects moments of the conditional distribution of the dependent variable at points other than the mean, then the assumption of mean-effects can be misleading and potentially result in costly mistakes from a policy maker’s point of view. To allow for the possibility that treatment and other determinants of household income affects may go beyond the conditional mean, we use quantile regression models [28]. This approach allows us to infer the extent to which treatment affects household welfare by altering the income distribution due to unobserved household specific factors.

We specify the regression model, based on data from each household *i*, is:
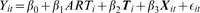
(1)where: *t = 1…5* denotes the period in which data was collected; *Y* denotes (logged) household gross income; *ART* is a dummy variable representing the treatment effect by indicating the last period when income data was collected after one year of the ART program; and **T** and **X** consist of time-variant and time-invariant control variables. The intercept term,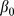, denotes the household’s income at the baseline, while 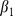 through 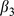 denote vectors of coefficients on the explanatory variables. The error term, 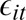, is assumed to be independently and identically distributed and uncorrelated with the explanatory variables.

The quantile regression model corresponding to equation 1 is specified as.
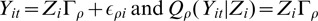
(2)where 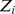 is the matrix of explanatory variables from equation 1; 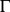 is a conformable matrix of coefficients; and 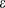 is the error term. 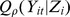 refers to the 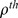 quantile of income conditional on Z. In equation 2, conditional quantiles are specified as a linear function of quantile specific parameters and specific values of covariates. The quantile specific parameters can be estimated using linear programming methods. We use a least absolute deviation (LAD) estimator and calculate standard errors through bootstrap methods.

#### Construction of variables

The data collected were used to construct a number of variables. For gross income, respondents reported wage labor and all quantities of goods produced and the quantities consumed or sold. The quantity of produced goods that are consumed represents the household’s subsistence production, whereas the quantity sold represents marketed products. We use consumed and sold quantities to calculate in-kind and cash income, respectively, by multiplying these quantities by a local prices. Not all households reported sales and prices for all products. Gross income for these households was calculated by using the mean of the price distribution from households that reported prices. Gross income is the sum of cash and in-kind income.

Principal-Component Factors were used to compute wealth indices from five wealth attributes as described in Table 1. In the analysis, the first principal factor explains 33% of the combined variance and is included as the wealth index (*wealth*) used here. We also calculated a knowledge score (*knowaids*) following a standardized test recommended by Kipp et al. [25], which tests the patient’s level of awareness of the basics of HIV/AIDS transmission. The knowledge score is the percentage of 12 Yes/No questions answered correctly.

**Table 1:** Description of independent variables and expected signs.

Variable names	Description	Expected sign
ART program impact (*ART*)	Dummy variable = 1 if the observation is associated with the last programme visit afterone year of ART program; = 0 if not	+
Time trend control (*time-trend*)	Time count variable indicating when each household started the program. The value ‘1′equals first quarter of 2006, while ‘10′ equals second quarter of 2008	+/−
Seasonal controls (*SQ1, SQ3, SQ4*)	Seasonal dummy variables = 1 if the observation is associated with quarters 1, 3 or 4; = 0 if not.The base case (i.e. omitted variable) is quarter 2, the short rainy season where incomeis expected to be lowest	+
Age of household head (*agehead*)	Age of household head in years	+
Average household education (*aveduc*)	Average years of education per adult living in the household (i.e. total years ofeducation obtained by all adults in the household divided by total number of adults)	+
Number of household adults (*hhadults*)	Number of household adults aged 10 to 65 years	+
Number of household dependents(*hhdeps*)	Number of household members aged 0 to 9 years and above 65 years	-
Household wealth index (*wealth*)	Household’s wealth index from −1.399 to 5.073; principal components include livestock,market value of durables, count of durables, home size, and land size	+/−
Household production expenses (*prodexpense*)	Household’s quarterly expenses on income-generating activities expressedin thousands of USh	+
Knowledge score of the patient (*knowaids*)	Knowledge score of the patient expressed as the percentage of correct answers from12 questions	+
Changes in Income Activities *(%changeforestY, %changecropY, %changelstockY, %changewageY, %changeremitY*)	Percent change in the portion of income derived from alternative livelihood activities(i.e. forests, crops, livestock, wages, and remittances) betweeneach data collection visit; income from small businesses is omitted to avoidover specification	+/−

The definitions of the variables employed, and their expected signs, are contained in Table 1. We expect the coefficient on *ART_i,_* to have a positive effect on income. We include in **T** two types of control variables – a time trend (*trend_time)* and seasonality (*SQ1-4*). Note that the time trend and seasonal effects are separately identified across the panel, as patient enrolment occurred over a 15-month period. The time trend controls for fluctuations in the macro-economic environment such as changes in overall demand and supply conditions. We have no expected signs of the time trend variables. Because crop demand and input purchases in subsistence economies may be seasonal, we also control for data being collected in each quarter. The base case is quarter 2 which represents the short rainy season where income is expected to be lowest. Therefore, we expect the other seasonal variables to have positive signs.

The control variables included in **X** consist of five groups. First, there are socio-demographic variables including age of household head (*agehead),* average education of adult household members *(aveduc),* number of household adults (*hhadults),* and number of household dependents *(hhdeps)*. For the first three variables, we expect a positive relationship with income as these factors have generally been found to increase the productivity of a household. For *hhdeps,* we expect a negative relationship as household resources can be spent more on support rather than production. Second, there are two socio-economic variables; a household wealth index (*wealth*) and quarterly production expenses (*prodexpense*). Though more wealth is typically associated with more income, households with HIV/AIDS patients may have drawn down their stocks of wealth to sustain incomes before treatments were started. Therefore, we have no expectation regarding the sign on this variable. We expect expenditures on production to have a positive relationship with income. Third, there are geographic variables that are represented by dummy variables for 10 parishes (not listed in Tables 1 and 2). Fourth, there is a disease awareness variable based on a knowledge score from the patient *(knowaids)*. The sign on this variable is expected to be positive. The more knowledge an individual under treatment has about HIV/AIDS, the better choices she/he can potentially make regarding treatment. Finally, we include a number of variables *(%change___Y*) that describe how much each of five income earning activities (See table 1), as a percentage of total income, changed between each data collection visit. We have no expectations regarding these signs.

**Table 2:** Regression results of impacts of treatment and control variables on household income levels (log of cash and in-kind income).

	Combined Income Groups		Low Income Group		Medium Income Group		High Income Group	
Variables^a^	Coefficients and (Std Error)	P-value	Coefficients and (Std Error)	P-value	Coefficients and (Std Error)	P-value	Coefficients and (Std Error)	P-value
*ART* (treatment variable)	0.301 (0.146)**	0.040	0.369 (0.200)*	0.063	0.325 (0.191)*	0.082	0.388 (0.173)**	0.018
*time-trend*	−0.018 (0.028))	0.532	−0.055 (0.052)	0.304	−0.002 (0.042)	0.964	−0.019 (0.046)	0.673
*SQ1*	0.262 (0.150)*	0.080	0.288 (0.199)	0.162	0.248 (0.215)	0.261	0.147 (0.197)	0.467
*SQ3*	0.386 (0.145)***	0.008	0.380 (0.226)*	0.113	0.224 (0.201)	0.282	0.164 (0.194)	0.440
*SQ4*	0.273 (0.143)*	0.056	0.212 (0.215)	0.336	0.096 (0.200)	0.63	−0.022 (0.205)	0.919
*agehead*	−0.012 (0.005)**	0.015	−0.015 (0.007)**	0.039	−0.013 (0.007)*	0.074	−0.012 (0.006)*	0.085
*aveduc*	0.065 (0.039)*	0.098	0.015 (0.059)	0.812	0.009 (0.075)	0.912	0.137 (0.077)*	0.082
*hhadults*	0.092 (0.030)***	0.003	0.071 (0.041)*	0.081	0.070 (0.037)*	0.05	0.077 (0.039)*	0.051
*hhdeps*	0.119 (0.041)***	0.004	0.205 (0.066)***	0.001	0.106 (0.055)*	0.063	0.079 (0.054)	0.157
*wealth*	0.170 (0.056)***	0.002	0.156 (0.101)	0.123	0.195 (0.104)*	0.056	0.095 (0.059)*	0.097
*prod-expense*	0.000 (0.000)	0.203	0.001 (0.001)	0.280	0.001 (0.001)	0.124	0.001 (0.001)*	0.058
*knowaids*	0.011 (0.005)**	0.049	0.010 (0.010)	0.288	0.009 (0.008)	0.262	0.012 (0.009)	0.160
*%changeforestY*	–	–	−0.009 (0.004)**	0.011	−0.008 (0.004)**	0.024	−0.007 (0.004)	0.118
*%changecropY*	–	–	−0.004 (0.003)	0.227	−0.001 (0.003)	0.686	−0.002 (0.002)	0.363
*%changelstockY*	–	–	−0.009 (0.004)**	0.054	−0.002 (0.004)	0.549	−0.002 (0.003)	0.531
*%changewageY*	–	–	−0.002 (0.004)	0.581	−0.001 (0.003)	0.695	−0.002 (0.003)	0.545
*%changeremitY*	–	–	0.002 (0.006)	0.698	0.002 (0.005)	0.637	−0.001 (0.005)	0.883
Constant	10.873 (0.559)***	0.000	10.633 (0.988)***	0.000	11.129 (0.826)***	0.000	11.695 (0.755)***	0.000
Observations	670		536^b^					
R-squared/Pseudo R-Squared	0.12		0.15		0.12		0.14	

a Variables are defined in Table 1.

b the number of observations for the quantile model is smaller than for the combined model because the quantile model includes the series of *%change* variables. We lose 134 observations due to calculating changes (as opposed to values associated with each data collection visit.

* significant at 10%;

** significant at 5%;

*** significant at 1%.

## Results

Table 2 contains the results of our estimations for a combined income groups model and for a quantile regression that indicates results for low, medium and high income groups. For all models, the treatment variable (*ART*) is significant. Because the dependent variable is logged, the estimated coefficients indicate that incomes of households, on average, increase somewhere between 30 and 39 percent after one year of the ART program. These results differ little across income groups.

There are also a number of control variables that are significant. For the time varying control variables, the time trend does not have a significant impact on income. For the seasonality controls, as expected, incomes tend to be higher relative to the short rainy season, particularly in the combined model. Seasonality is not generally significant in the quantile results.

For the socio-demographic control variables, the age of the household head is statistically significant, but contrary to our expectations has a negative sign. We suspect that many of the household heads in our sample, with an average age of 43 years, are beyond their most productive years. At any rate, the size of the coefficient indicates that this variable does not have a great impact on incomes (i.e. each year of age only decreases income by approximately 1.5%). The average education variable is significant in the combined model and for the high income group where a one year increase in the average years of education in the household increases income by 14%. It appears as though education is most effective in influencing income for those that are relatively well-off. The number of adults in a household is also positively related to income, with each adult increasing income by approximately 8%, irrespective of income group. An unexpected result is a positive relationship between the number of household dependents and income, particularly in the lower income groups. Low income households appear to be using dependents to increase their income by large amounts (i.e. 21%), while dependents in medium income households increase incomes by a smaller amount (i.e. 11%). The coefficient for high income households is small and insignificant.

Regarding the socio-economic control variables, we see that for the combined income groups, an increase in the wealth index by one point increases income by 17%. Wealth effects are also generally significant across income levels, with income increases varying between 10 and 20%. Production expenses are generally not significantly influencing income, except for the high income households, where each one thousand USh spent as a cash input increases income by 0.1.

For the geographic control variables (not reported), there are some significant parish effects, which sometimes vary across households. As expected, knowledge of HIV/AIDS is positively associated with increased incomes, except for low income households, where a 1% increase in the knowledge score increased incomes in the combined model, increasing incomes by approximately 1%. This variable is not, however, significant in the quantile results. Finally, changes in the types of activities that households pursue may significantly influence incomes. For all income groups, a 1% increase in forest income leads to an approximate 1% decrease in overall income, though this effect is slightly below the 10% level of significance for high income groups. There is also a similar result regarding livestock income and low income households.

## Discussion and Conclusions

Despite convincing evidence regarding the health benefits of ART programs in developing countries, funding for such initiatives has been waning. In this paper, we contribute to the case for investments in ART programs by estimating the impacts of treatments on household incomes. Results indicate that treatment to an individual member in a household increases income for the entire household (across households with different income levels) by approximately 30% to 40%. Though the importance of such an increase in income is difficult to assess, the increase is, at least intuitively, impressive. For example, each investment in an individual’s primary education in Uganda has been estimated to increase their wages by approximately 10% [29]. More generally, increasing incomes in Uganda from $1.25 per day to $2.00 per day (an increase of 60%) would pull 38% of the population out of poverty [30].

In estimating the impacts of this ART program, we control for a number of variables, including temporal, geographic, socio-demographic, and socio-economic considerations. The impacts of these control variables on incomes in recovering HIV/AIDS households disclose a number of policy implications for development strategies. First, we find that human capital, in the form of education, plays a critical role in complementing the rising income of recovering households. For the overall sample a one year increase in the average education of adults in a household increases income in recovering households by 7%. For the wealthiest households, whose resources may make them better able to realize investments in human capital, the impact of education on income is 14%. These results suggest strong complementarities between human capital and other forms of wealth. Indeed, the wealth variable in the combined model indicates that wealth plays a significant and positive role in increasing the incomes of households recovering from HIV/AIDS.

Household composition is also important in influencing incomes in recovering households. Though the role of adults is uniformly important across income classes, the role of children differs. For the poorest households an additional child increases household income by 20%. In medium income households, the corresponding increase is 10%, while the increase is insignificant in the richest households. The role of children in households seems to change as income increases. In poorer households, children are part of the labor force contributing to income. In contrast, richer households seem to be using their children as a source of investment in education for future gains.

Estimating quantitative effects of HIV/AIDS treatments on household welfare is challenging. In this context, one evaluation method, that bypasses problems of causal interpretation, is a randomized control trial. However, in our research situation, for ethical reasons, we were not able to secure a sample of HIV/AIDS infected people who were not treated. Accordingly, we settled for a quasi-experimental approach where the treatment effects were identified temporally (i.e. before and after the treatment). However, such an approach required us to control for temporal factors and household specific characteristics that were thought to influence incomes of households. In sum, in the absence of being able to conduct a randomized control trial, we believe that we have effectively purged conflating effects of factors that could bias our results. Therefore, we are confident that our approach provides reliable estimates of the effect of HIV/AIDS treatments on income levels.

Overall, our results suggest a strong case for investing in ART programmes and further point to the importance of recognizing the heterogeneity in complements needed for effective programs and the general development of these local economies. As households recover from HIV/AIDS, government programmes which relieve human and physical capital constraints, faced most severely by the poorest households, would likely be strong complements to improving health and producing subsequent increases in income.
